# School amalgamation and wellbeing for LGBTQ+ students: A scoping review protocol

**DOI:** 10.1371/journal.pone.0318681

**Published:** 2025-02-12

**Authors:** Luke Slattery, Daragh Bradshaw, Sarah Jay, Aisling T. O’Donnell, Lynn Fenton

**Affiliations:** 1 Centre for Social Issues Research, Department of Psychology, University of Limerick, County Limerick, Ireland; 2 Health Research Institute, University of Limerick, County Limerick, Ireland; Woldia University, ETHIOPIA

## Abstract

**Introduction:**

School amalgamations, or the merging of two or more pre-existing schools, are typically conducted in response to resource constraints. While merging existing schools can be financially beneficial, the wellbeing of marginalised students, such as those who identify as LGBTQ+ , may be at risk. Evidence on the impact that school amalgamation may have for LGBTQ+ students’ wellbeing has not been consolidated in a review. The proposed scoping review aims to identify empirical studies which measure the impact of school amalgamation on the wellbeing of LGBTQ+ students, to document the methodologies they use, and to identify challenges faced by LGBTQ+ students in amalgamated schools.

**Methods:**

This review will be conducted in line with the JBI Manual for Evidence Synthesis and PCC framework. Searches will be conducted in multidisciplinary databases and relevant citations exported to Covidence. Articles will be screened against inclusion and exclusion criteria by two independent reviewers.

**Results:**

Relevant studies will be charted and synthesised for inclusion in the final scoping review. Particular attention will be given to the scope of existing literature relating to the review question, methodological trends, and areas for further study.

## Introduction

School amalgamation refers to the merging of two or more pre-existing schools into one educational setting [1]. While amalgamation can be an effective resource management strategy [2], we have limited understanding of the long-term impact these mergers have on students, particularly those who are marginalised. When schools merge, students must learn to navigate an unfamiliar educational and social environment [3]. Divisions between student groups during this transition can result in negative peer interactions and bullying [3]. These periods of social adjustment may be especially challenging for marginalised students [3]. One such marginalised group are those who identify as lesbian, gay, bisexual, transgender, or other sexual and gender minorities (LGBTQ+). LGBTQ+ youth are regularly associated with both negative mental health outcomes [4–6] and barriers to school-based social integration [7,8]. Given the unique, and potentially hostile, social environment that the transitional stages of school amalgamation may pose [3], the challenges faced by LGBTQ+ students may be exacerbated, resulting in negative consequences for their wellbeing.

Poorly implemented organisational amalgamations can be traumatic for those involved [9]. Traumatic experiences during adolescence have been shown to undermine social, health, and educational trajectories, particularly for young people with marginalised identities [10,11]. These potential threats to long-term wellbeing underscore the importance of clarifying how school amalgamations are currently conceptualised in relation to wellbeing, consolidating findings, and charting data relating to outcomes for LGBTQ+ students. The proposed scoping review therefore aims to retrieve and consolidate empirical evidence relating to the impact of school amalgamations on wellbeing for LGBTQ+ students, the challenges faced by LGBTQ+ students in amalgamated schools, and the methodologies relevant studies use. The protocol for this review has been structured in reference to existing peer-reviewed scoping review protocols and Ghezzi-Kopel and Porciello’s evidence synthesis template, which incorporates guidance from interdisciplinary librarians with the aim of enhancing dissemination and strengthening synthesis methodologies [12–14].

A preliminary search of the literature was conducted in October of 2024. No systematic or scoping reviews, either final or registered report protocols, relating specifically to the wellbeing of LGBTQ+ students in amalgamated schools were identified. Similarly, no existing protocols for reviews relating to the proposed topic were identified via Open Science Framework or PROSPERO. One rapid scoping review relating to school amalgamations more generally was identified [1]. This existing review offers a beneficial overview of administrative, financial, academic, social, and geographic considerations of amalgamations [1]. While the authors discuss educational disadvantages and rural-urban disparities, further evidence consolidation is needed to understand the marginalisations experienced by LGBTQ+ youth and their link to wellbeing as an outcome. Additionally, this review did not include studies published prior to the year 2000 [1]. The proposed scoping study will examine relevant papers which were potentially omitted by this rapid review while providing a much-needed psychological approach to understanding amalgamation and wellbeing for LGBTQ+ students.

Preliminary searches suggest that research on marginalised students’ experiences of amalgamation and wellbeing is rare and spread across disciplines, making it difficult for researchers and those enacting school mergers to access and supporting the use of scoping review methods. As outlined by Arksey and O’Malley [15], scoping studies are conducted to examine the extent of existing research, identify research gaps, and to both summarise and disseminate relevant findings [12]. The proposed review aims to improve the accessibility of literature relating to LGBTQ+ students’ experiences of amalgamation and wellbeing. The final published review will allow both researchers and key stakeholders in existing and future amalgamations to easily access an overview of studies, identify important findings, and develop research questions from gaps that may emerge.

## Methods

Methodological guidance for this review has been drawn from Chapter Ten of the JBI Manual for Evidence Synthesis [16]. The JBI Manual for Evidence Synthesis has been chosen as it provides comprehensive, evidence-based guidance to conducting and reporting systematic scoping reviews. This manual exists in an online format and is updated regularly [17]. By using this structured, accessible resource, we also enhance the replicability of our methods. As recommended by JBI, the Population/Concept/Context (PCC) framework has been used for constructing our review questions and search strategies [18]. This framework communicates clear objectives and inclusion criteria when included at the protocol stage while ensuring our eventual data extraction is relevant to the review question [18]. Any deviations from the methods set by JBI will be clearly reported and justified in the final scoping review.

### Review questions

The review questions for the proposed scoping review, as developed in line with the PCC framework, are as follows:

RQ1: What empirical literature is available which documents the impact of school amalgamation on the wellbeing of LGBTQ+ students?
RQ2: What methods have been used to measure the wellbeing of LGBTQ + students in amalgamated schools?
RQ3: What are the challenges faced by LGBTQ+ students in amalgamated schools, as recorded in current literature?

### Definitions

As advised in Ghezzi-Kopel and Porciello’s evidence synthesis template [13,14], definitions for key terms identified using the PCC framework are provided below.

#### School amalgamation.

The words ‘amalgamation’ and ‘consolidation’ are used interchangeably in the literature. These terms are used to refer to the merging of two or more pre-existing schools into a single school or educational set-up [1]. Amalgamations have been described as both a positive resource management strategy and a threat to the livelihood of school communities [19,20]. The rationale given for enacting amalgamations includes improved resource provision for small rural schools, legislative changes and fiscal challenges within school boards, and demographic changes amongst local school-aged children [9,21,22]. For this review, changes to school set-ups are considered amalgamations where a minimum of two schools which previously operated independently are brought together to form a single institution.

In their rapid review of regional and rural school amalgamations, Eacott and Freeborn include evidence from studies on both school-level and district-level consolidations [1]. School districts refer to geographical boundaries which dictate the governance and administration of schools within a given area. The use of school districts is observed primarily in the United States [1,23,24]. While district consolidations share similarities with school consolidations in terms of their rationale, such as resource management and governance changes, fewer comparisons can be drawn at the student level. As district consolidations do not inherently involve the merging of school populations, studies focusing on district-level mergers will not be considered as meeting the definition criteria of school amalgamation. Studies which address outcomes of district mergers may still be included if these district mergers have resulted in subsequent school amalgamations.

#### Students.

For this review, students are defined as those enrolled in primary or secondary education. While the term ‘student’ can more broadly reflect those enrolled in third level or other forms of education, these definitions are beyond the scope of our review questions. There is no age parameter set in defining student populations as age ranges are expected to be uniform across childhood and adolescence given their educational stage.

#### LGBTQ+ identification.

For the purpose of this review, definitions of sexual or gender minority identities are derived from the American Psychological Association style guide [25]. The LGBTQ+ acronym is used when simultaneously referring to multiple sexual and gender minority groups [25]. Sexual orientation refers to both a person’s attraction to another person and the social categorisations that arise from this attraction [25]. A person may be defined as holding a minority sexual identity if their sexual orientation is not heterosexual. A person may be defined as holding a minority gender identity if their gender does not align with their sex assigned at birth [25]. Any variations in accounts of LGBTQ + identification among students will be reported in the final scoping review.

#### Wellbeing.

To address the impact of amalgamation from a psychological perspective, wellbeing is defined as both general perceptions of life satisfaction and mental health outcomes. General wellbeing and life satisfaction may relate to concepts such as quality of life, self-esteem, and happiness. Mental health outcomes relate more specifically to psychological distress, such as anxiety, depression, and other mood disorders.

### Objectives

This review aims to uncover the scope of research relating to school amalgamation and wellbeing for LGBTQ+ students as well as the methods they employ. We will identify both patterns and gaps in research topics and themes, observed populations, data sources (i.e., primary or secondary analyses) and methodological approaches. In doing so, a consolidated body of literature will be available to both researchers and key stakeholders in ongoing and future school amalgamations.

### Eligibility criteria

The JBI Manual for Evidence Synthesis has been used in conjunction with the PCC framework when defining inclusion and exclusion criteria for this review [16]. As suggested by Ghezzi-Kopel and Porciello [14], numbered lists of study inclusion and exclusion criteria (and, where relevant, their alignment with the PCC framework) are provided below.

#### Inclusion criteria.

1. Population: School-aged students who identify as LGBTQ+ and are involved in the amalgamation of primary or secondary schools.
2. Concept: The amalgamation of at least two pre-existing primary or secondary schools into one educational setting, and the impact of such amalgamations on the wellbeing of marginalised students.
3. Context: Primary or secondary schools which conduct in-person teaching and learning.
4. Published, peer-reviewed articles which contain original research, meta-analyses, systematic reviews, experimental research, case studies, or other quantitative, qualitative, or mixed-method approaches.
5. Articles must be published in peer-reviewed journals, either open-access or accessible through the related institution’s library agreements and repositories.
6. Due to language limitations within the research team, articles must be published in English.

#### Exclusion criteria.

1. Population: Students outside of primary or secondary-level education (e.g., those studying in third-level institutions), or those who do not identify as LGBTQ+ , or those who do not attend amalgamated schools.
2. Concept: Studies which focus on school amalgamation, but do not consider wellbeing-related outcomes or LGBTQ+ identification, or those which address similar concepts (e.g., school-district amalgamations) without reference to the amalgamation of actual schools.
3. Context: Third-level institutions, schools which solely employ online or otherwise distant teaching methods, schools which previously amalgamated but whose entire student population at the time of study had not enrolled until post-amalgamation.
4. Non-peer-reviewed articles or otherwise non-empirical studies, including book reviews, opinion pieces, theses, conference abstracts, and editorial letters.
5. Articles which are not openly accessible to the research team and/or contained in non-peer-reviewed journals or research repositories.
6. Studies published in languages other than English.

### Information sources

Multidisciplinary databases have been chosen for their relevance to the topic of interest and related research questions. The databases to be searched are APA PsycArticles, APA PsycInfo, British Education Index, Education Resources Information Center (ERIC) and Scopus. EBSCOhost will be used to facilitate these searches where applicable.

### Search strategy

Initial limited searches of ERIC and APA PscyInfo were undertaken to assist the development of a full scoping search strategy. Search terms were selected in accordance with the PCC framework, with one population category (*LGBTQ+ students*), two concept categories (*amalgamation*, *wellbeing*) and one context category (*primary and secondary schools*) respectively. The key words contained in the titles and abstracts of relevant articles and their index terms were used in developing a search strategy for a full scoping search. The reference lists of these sources were also screened to identify potential synonyms and further keywords.

The final scoping search strategy for this review has been created in consultation with a librarian who specialises in education and health sciences research. To ensure results are relevant to our review questions, terms relating to amalgamation and schools will be searched within titles and abstracts. As findings relating to LGBTQ+ identification and wellbeing may be contained in papers where these concepts were not of primary focus, these terms will be searched within the full text of articles. Finally, where databases such as ERIC allow for searches within descriptor categories, key terms will be searched under relevant category headings (e.g., ‘Mergers’, ‘Organizational Change’, ‘Consolidated Schools’). A sample search strategy for use in ERIC and APA PsycInfo via EBSCOhost can be viewed in S1 Appendix A.

### Study records

#### Data management.

Covidence will be used to manage data and citations throughout the review process. Articles identified using our search strategy will be imported to Covidence for title and abstract screening, duplication removal, full-text extraction, and record keeping. To ensure the replicability of our search and selection processes, data will be reported using the Preferred Reporting Items for Systematic reviews and Meta-Analyses extension for Scoping Reviews checklist (PRISMA-ScR) [26]. A sample of this checklist can be viewed in S2 Appendix B.

#### Selection process.

After relevant exclusion criteria are applied (e.g., excluding non-English papers), all remaining citations will be transferred to Covidence and duplicates removed. Titles and abstracts will be screened by two independent reviewers against the inclusion criteria for the review. Potentially relevant studies agreed upon by both reviewers will be retrieved in full. The full text of these studies will then be assessed. Reasons for exclusion of full text studies will be systematically recorded and reported in the final scoping review. Any disagreements that arise between the reviewers during study selection will be resolved through discussion and, if necessary, the input of an impartial third reviewer. Search results will be reported both narratively and in a Preferred Reporting Items for Systematic Reviews and Meta-analyses (PRISMA) flow diagram [27]. A sample diagram is provided in S3 Appendix C.

#### Data charting.

As extraction tools within Covidence are typically used when reviewing clinical trials via the PICO framework, Microsoft Excel will be used for data charting. A data charting instrument has been created using recommendations from both Arksey and O’Malley’s influential work on scoping review methodologies and JBI’s evidence details, characteristics and results extraction instrument [15,16]. These established instruments have been tailored for relevancy to the review questions. The criteria for data charting are as follows:

1. Citation details (author, publication year, journal name).
2. Study descriptors (location, year conducted).
3. Participants (age, school year, total sample size, LGBTQ+ sample size).
4. Context (details of school amalgamation).
5. Study aims.
6. Methodology (e.g., surveys, focus groups, mixed methods).
7. Measurements of wellbeing.
8. Relevant results.

These criteria may be modified with the addition of previously unidentified themes or points of interest should they emerge during data screening. Any modifications will be reported in the final scoping review. All extracted data related to our findings will be made available through supplementary material published alongside the final study.

### Data synthesis

Results of our search strategy will be synthesised using a PRISMA flow chart diagram [27] and accompanied by a brief narrative description. Relevant data will be reported both narratively and in tables. Findings will initially be categorised based on the extraction criteria defined above, then synthesised into overarching themes relating to the scope of current literature, prominent methodologies, and results pertaining to marginalisation and wellbeing among LGBTQ+ students attending amalgamated schools. A full table which aligns with the journal’s data availability policy will be provided with citation details for the identification of included and excluded papers.

## Supporting information

S1 Appendix A Sample search strategy for use through ERIC and APA PsycInfo via EBSCOHost (search results as of November 5th, 2024). (TIF)

S2 Appendix B PRISMA-Scr checklist. (TIF)

S3 Appendix C PRISMA flow diagram. (TIF)
