# Supplementary figures and images for: School amalgamation and wellbeing for LGBTQ+ students: A scoping review protocol
Source: PLoS One. 2025 Feb 12;20(2):e0318681. doi: 10.1371/journal.pone.0318681 (PMC11819577; doi:10.1371/journal.pone.0318681)

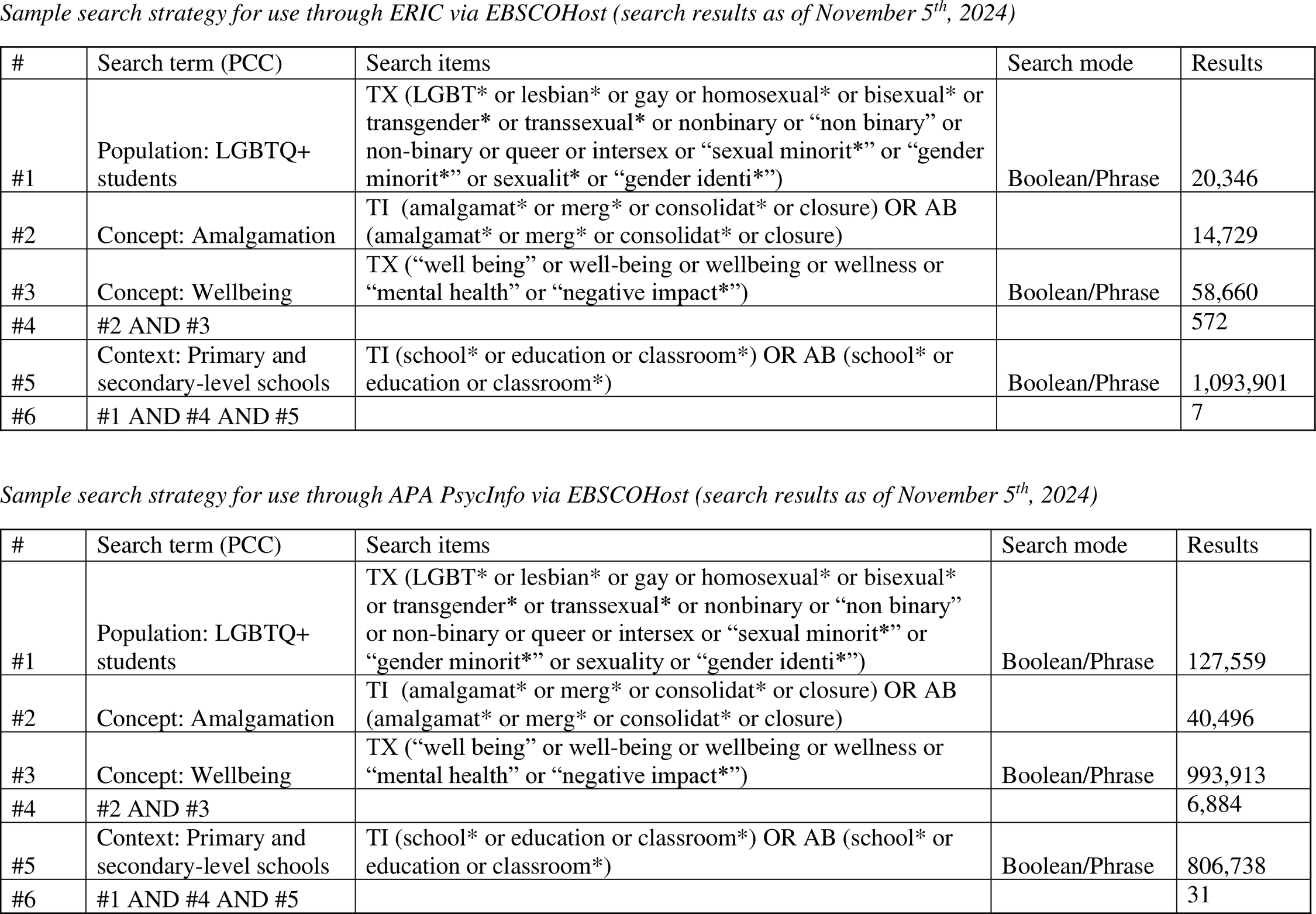

Supplement: S1 Appendix A — (TIF) [file pone.0318681.s001.tif]

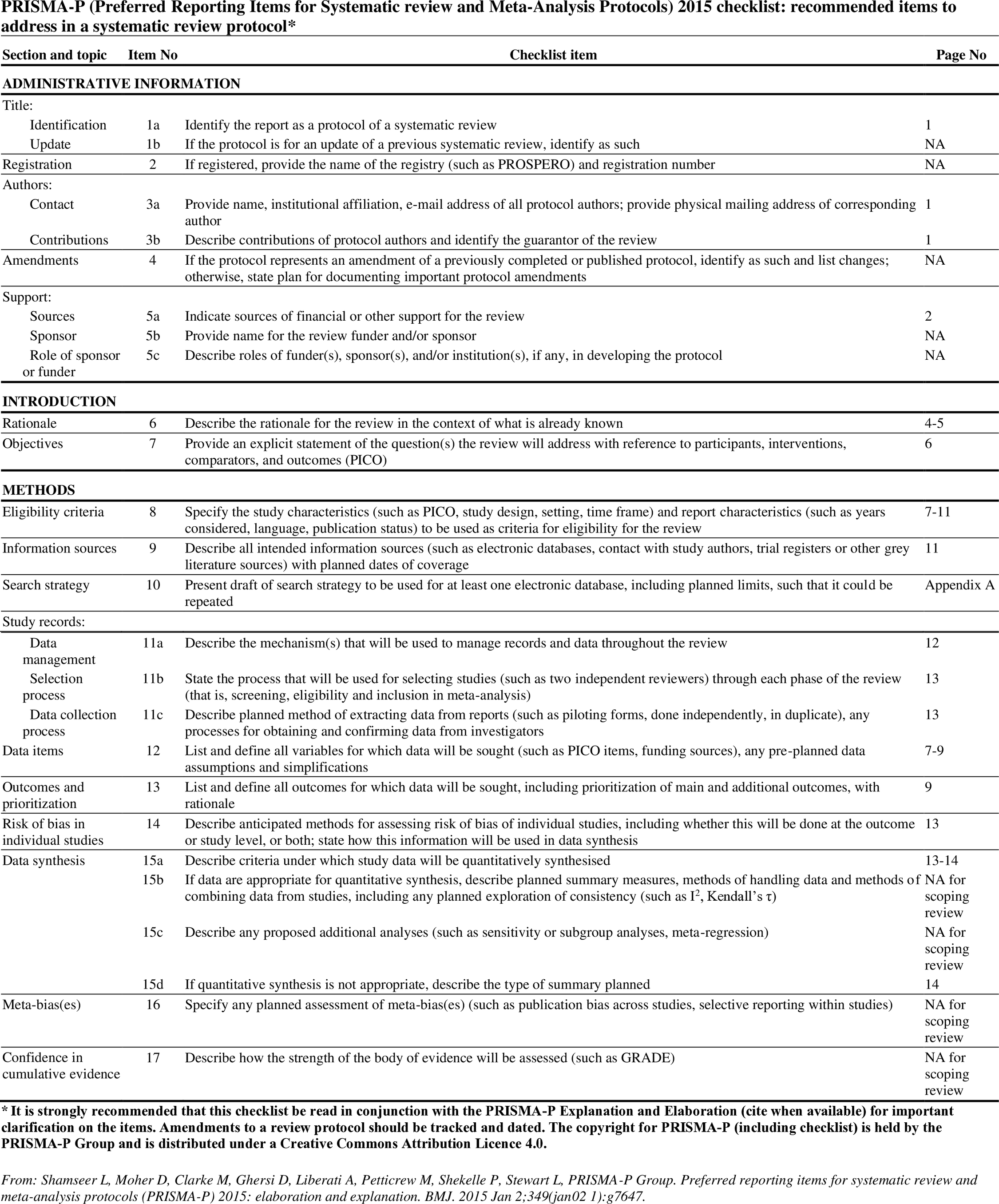

Supplement: S2 Appendix B — (TIF) [file pone.0318681.s002.tif]

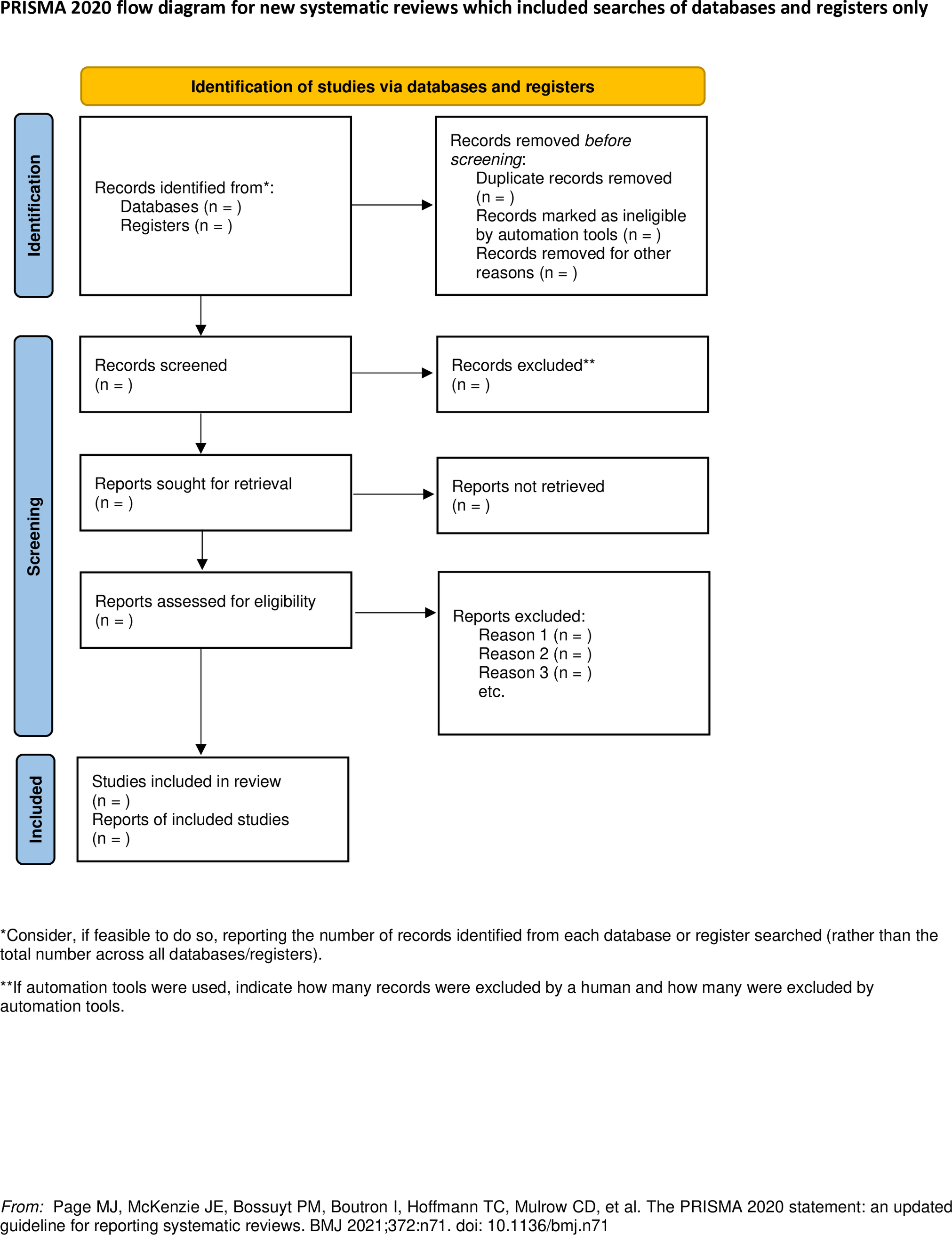

Supplement: S3 Appendix C — (TIF) [file pone.0318681.s003.tif]
